# Wirsung atraumatic rupture in patient with pancreatic pseudocysts: a case presentation

**DOI:** 10.1186/s12876-018-0781-3

**Published:** 2018-04-23

**Authors:** Martino Gerosa, Marco Chiarelli, Angelo Guttadauro, Matilde De Simone, Fulvio Tagliabue, Melchiorre Costa, Sabina Terragni, Ugo Cioffi

**Affiliations:** 1 Department of Surgery, ASST Lecco, Via dell’Eremo 9/11, 23900 Lecco, Italy; 2 Department of Surgery, University of Milan-Bicocca, Istituti Clinici Zucchi, Via Zucchi 24, 20900 Monza, Italy; 3 Department of Surgery, University of Milan, Via F. Sforza 35, 20122 Milan, Italy

**Keywords:** Pseudocyst, Jaundice, Pancreatic duct rupture, Acute pancreatitis, Pancreatic cystic mass

## Abstract

**Background:**

Pancreatic duct disruption is a challenging condition leading to pancreatic juice leakage and consequently to pancreatic fluid collections. The manifestations of pancreatic main duct leak include pseudocysts, walled-off necrosis, pancreatic fistulas, ascites, pleural and pericardial effusions. Pseudocyst formation is the most frequent outcome of a pancreatic duct leak.

**Case presentation:**

We describe a case of a 64-year old man with large multiple pancreatic cysts discovered for progressive jaundice and significant weight loss in the absence of a previous episode of acute pancreatitis. Computed tomography scan showed lesion with thick enhancing walls. The main cyst dislocated the stomach and the duodenum inducing intra and extrahepatic bile ducts enlargement. Magnetic resonance cholangiopancreatography revealed a communication between the main pancreatic duct and the cystic lesions due to Wirsung duct rupture. Endoscopic ultrasound guided fine needle aspiration cytology did not show neoplastic cells and cyst fluid analysis revealed high amylase concentration. Preoperative exams were suggestive but not conclusive for a benign lesion. Laparotomy was necessary to confirm the presence of large communicating pseudocysts whose drainage was performed by cystogastrostomy. Histology confirmed the inflammatory nature of the cyst wall. Subsequently, the patient had progressive jaundice resolution.

**Conclusion:**

Pancreatic cystic masses include several pathological entities, ranging from benign to malignant lesions. Rarely pseudocysts present as complex cystic pancreatic lesions with biliary compression in absence of history of acute pancreatitis. We describe the rare case of multiple pancreatic pseudocysts due to Wirsung duct rupture in absence of previous trauma or acute pancreatitis. Magnetic resonance showed the presence of communication with the main pancreatic duct and endoscopic ultrasound fine needle aspiration suggested the benign nature of the lesion.

## Background

Pancreatic pseudocysts are the consequence of a pancreatic duct disruption, especially as a result of acute necrotizing pancreatitis. They complicate acute pancreatitis episodes in 5%–15% of cases [1]. Wirsung rupture is usually associated with chronic pancreatitis or trauma [2]. The most frequent clinical presentation of pancreatic pseudocyst is abdominal pain [3], while obstructive jaundice is uncommon [4]. Pancreatic cystic mass diagnosis, in the absence of previous history or risk factors for pancreatitis, could be demanding.

We describe a case of multiple pancreatic pseudocysts presenting with jaundice in a setting of atraumatic Wirsung duct rupture without symptoms of acute pancreatitis.

## Case presentation

A 64 year old man presented to our emergency department for 2 weeks jaundice and 10 kg weight loss in the last two months. The patient did not report previous abdominal trauma, abdominal pain or history of alcohol abuse.

An outpatient abdominal ultrasound (US) showed a complex large cystic mass in the epigastric area. The serum level of total bilirubin was 7.5 mg/dL, direct bilirubin 6.0 mg/dL, lipase 881 U/L. The level of serum carcino-embryonic antigen (CEA) was 0.9 ng/mL (normal range 0–5.0 ng/mL), carbohydrate antigen 19–9 (Ca 19–9) 17.4 U/mL (normal range 0–37 U/mL), carbohydrate antigen 125 (Ca 125) 58.9 U/mL (normal range 0–35 U/mL), chromogranin A (CgA) 1.6 nmol/L (normal range 0.5–3.0 nmol/L) and neuron specific enolase (NSE) 14.3 μg/L (normal range 0–12.5 μg/L).

**Fig. 1:**
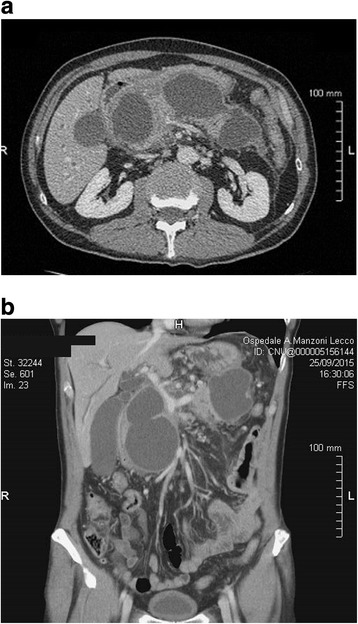
**a-b** CT scans showed multiple defined hypodense lesions with peripheral contrast enhancing pancreatic areas compatible with cystic lesions. The main one involved the head of the pancreas and measured 12 cm in diameter

Triphasic multidetector computed tomography (CT) scan showed multiple pancreatic cystic lesions with thick enhancing walls and homogeneous liquid content (Fig. 1a-b). The main one, measuring 12 cm in diameter, involved the head of the pancreas. The lesion dislocated the stomach and the duodenum and determined intra and extrahepatic bile ducts enlargement. CT scan also showed a thickening of the mesentery root and pararenal spaces with enlargement of several loco-regional lymph nodes. Other two large (10 cm and 9 cm respectively) cystic lesions were identified in the body and the tail of the gland. Moreover CT scan showed a communication between the three cystic lesions.

**Fig. 2:**
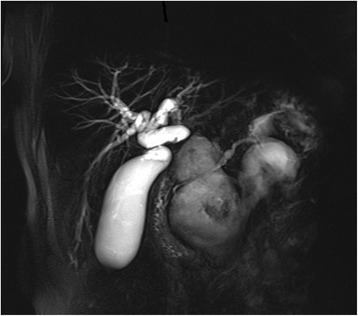
MRCP scan confirmed multiple confluent pancreatic cysts. It revealed a communication between the main pancreatic duct and the cystic mass suspected for Wirsung duct rupture in the pancreatic tail

Magnetic resonance cholangiopancreatography (MRCP) scan confirmed the presence of large multicystic mass and revealed a communication between the main pancreatic duct and the complex lesion in the tail of the gland, suspected for Wirsung duct rupture (Fig. 2). MRCP also excluded the presence of a dilatation of the main pancreatic duct.

**Fig. 3:**
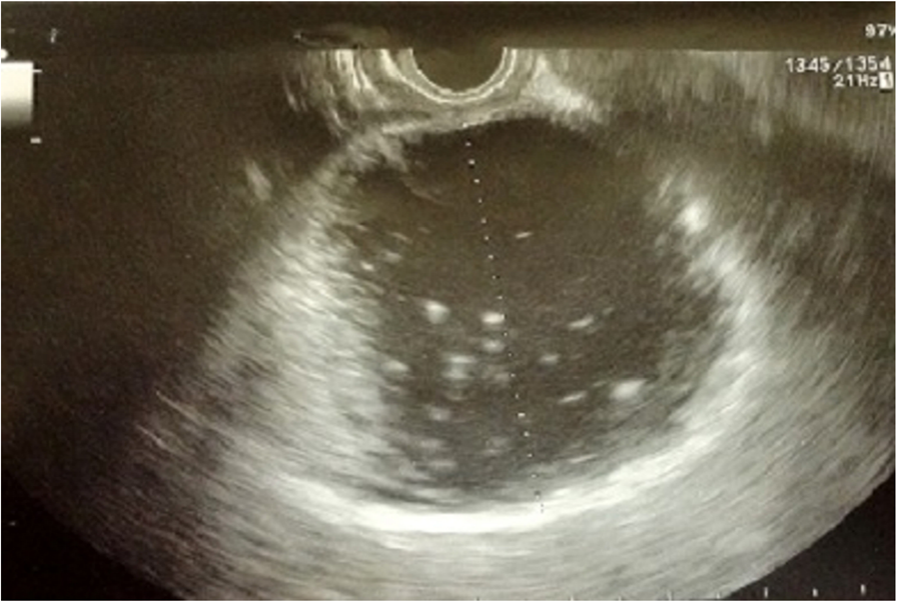
EUS showed pancreatic head cystic lesion with thickened walls and serous content

Endoscopic ultrasound (EUS) also confirmed the presence of a multicystic pancreatic lesion. Fine-needle aspiration cytology (FNAC) showed the presence of granulocytes, histiocytes and the absence of neoplastic cells (Fig. 3). Fluid amylase concentration was 8977 U/L; CEA was < 5 ng/mL. Preoperative exams were suggestive but not conclusive for a benign lesion. Consequently an explorative laparotomy was necessary for definitive diagnosis and appropriate treatment.

**Fig. 4:**
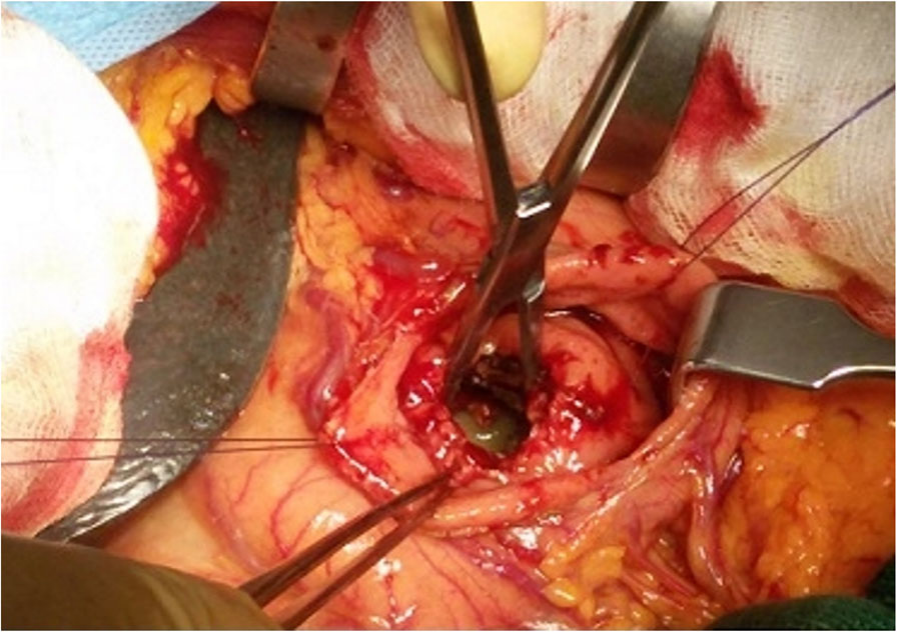
Surgical view. After anterior and posterior gastric walls were opened pancreatic pseudocyst appeared and its cavity entered for drainage

Laparotomy showed diffuse mesocolic and omental calcifications with steatonecrosis as occurring in pancreatitis. A large cyst originating from the head of the pancreas dislocated the duodenum laterally and the stomach anteriorly (Fig. 4). A sampling of the liquid content of the cyst confirmed a serous fluid rich in amylase (3132 U/L). Indeed frozen section of the cyst wall pointed out the inflammatory nature of the lesion. Main cyst drainage was achieved by hand sewn cystogastrostomy.

After the procedure we observed jaundice resolution, cholestatic indexes improvement and oral intake resumption. Postoperative CT scan showed significant down-sizing of the pseudocysts. The patient was discharged 15 days after the operation.

## Discussion and conclusion

Pancreatic duct disruption is accompanied by pancreatic juice leakage and consequently determines pancreatic fluid collections (PFCs).

The clinical manifestations of pancreatic main duct leak include pseudocysts, walled-off necrosis (WON), pancreatic fistulas, ascites, pleural and pericardial effusions [5]. According to the revised Atlanta classification, pseudocysts and WON are the late results (> 4 weeks after pain onset) of post-pancreatitis collections [5, 6]. Pseudocyst is the most frequent outcome of a pancreatic duct leak. WON is secondary to a focal pancreatic necrosis and is characterized by the presence of necrotic debris in the collection, while pseudocyst content is serous [6].

Disconnected duct syndrome (DDS) is a rare condition characterized by a pancreatic leak with a complete transection of the main pancreatic duct in the distal portion of the gland. It generally occurs as a result of severe acute pancreatitis with pancreatic necrosis and can be seen in 16% of these patients [7].

CT and MRCP scan are the pivotal imaging exams in patients presenting with a PFC [5]. CT frequently overestimates the fluid component of a collection and may misdiagnose WON as a pseudocyst [8]. MRCP could be used as a substituted of endoscopic retrograde cholangio-pancreatography (ERCP) as it can identify an active leak from a pancreatic duct [9]. EUS can be particularly helpful in identifying necrotic debris within the fluid collections [5]. EUS-FNA can be useful in differential diagnosis of cystic lesions by collecting fluid for cytology, amylase and CEA [10–12]. Pseudocyst content is usually characterized by high amylase concentration and low CEA levels. In these patients the fluid of the cyst usually contains inflammatory cells on cytologic evaluation [5]. Nevertheless cytology and fluid analysis are characterized by some limitations. In a large series FNAC showed high specificity (83%) but low sensibility (34%) for mucinous cystic lesions [10]. Only cyst fluid CEA concentration (cutoff value = 192 ng/mL) was associated with remarkable specificity and sensibility (83% and 75% respectively) for mucinous lesions [10]. Indeed, amylase concentration on cyst fluid may be elevated not only in pseudocysts but also in neoplasms with pancreatic ducts communication [13].

Our patient had no risk factors, history or signs of acute pancreatitis. No previous abdominal trauma was reported. Symptoms of presentation were jaundice onset and significant weight loss. Abdominal pain is the most frequent symptom of presentation of a pseudocyst [3]. Jaundice and weight loss occur more frequently in pancreatic neoplasms. In a large retrospective analysis, jaundice and weight loss account respectively for 3.9 and 18.6% of symptoms presentation of a pseudocyst [14]. Indeed patients developing pseudocysts are rarely asymptomatic for pancreatitis, making our diagnosis more challenging. Mucinous cystic neoplasm (MCN) and intraductal papillary mucinous neoplasm (IPMN) were considered as alternative diagnosis [15].

MCNs are thick-walled macrocystic tumors without ductal system communication [16–18]. Large cystic lesions detected on CT scan were coherent with MCN but MRCP findings excluded this diagnosis. IPMN was considered due to the presence of a communication between the cysts and the pancreatic ducts; however the dilation of Wirsung duct, characteristic of IPMN type main duct, was absent.

Nevertheless the lack of neoplastic cells on EUS-guided FNAC and the high concentration of amylases made the diagnosis of a benign cyst more probable than a malignant lesion [16].

Management of PFCs is conservative in case of small collections, small duct defects and in the absence of ductal obstruction downstream consequent to duct disruption [6]. Large ductal disruptions with downstream obstruction require operative management. Nowadays only symptomatic PFCs are recommended for drainage [6]. In our case the presence of jaundice, upper gastrointestinal compression and large size of pseudocysts required drainage. Currently the drainage of a pseudocyst can be performed with a surgical or endoscopic technique: in the last decades endoscopic treatment has become the approach of choice [19].

Surgical cystogastrostomy is usually performed through an anastomosis between the lumen of the cyst cavity and the stomach [20]. Surgical drainage is an efficacious therapy. After drainage, pseudocyst recurrence rate is 2.5%–5%, but the rate of complications can reach 30% [21].

Endoscopic treatment modalities are transmural drainage, transpapillary drainage and pancreaticoduodenostomy or gastrostomy.

Initial studies comparing surgical to endoscopic cystogastrostomy showed equivalent pseudocyst resolution and comparable complication rates [22, 23]. However, the improvement of minimally-invasive skills made the endoscopic technique the preferred initial approach. More recent studies concluded that two techniques yielded similar technical success and complication rates but endoscopic treatment had shorter length of hospital stay and lower hospital cost [24–26]. Furthermore a Cochrane review comparing surgical and endoscopic treatment modalities concluded that further studies are required on this topic [27]. A recent analysis concluded that the optimal treatment for pancreatic pseudocyst is still controversial, in the absence of meta-analysis comparing the two approaches [25].

We performed surgical exploration for three reasons. Firstly, the peritoneal exploration and the frozen section definitively could exclude the tumor nature of the cystic lesion. Moreover, the surgical drainage allowed a faster biliary and stomach decompression. Finally, a reduced experience in pseudocysts endoscopic drainage with EUS guidance at our institution made surgical treatment more reliable.

Retrospectively, in our case, the more reliable explanation of the multiple pseudocysts origin was a rupture of the main pancreatic duct secondary to an asymptomatic focal necrotic pancreatitis of the tail of the gland. The complex cystic mass was later revealed due to gastric and biliary tract compression.

Pancreatic pseudocysts generally complicate acute and symptomatic episodes of pancreatitis. In our case the presence of a complex cystic pancreatic lesion and Wirsung duct rupture in the absence of a previous episode of acute pancreatitis or abdominal trauma made the diagnosis challenging. In such cases, CT, RM and EUS are essential for suspecting malignant lesions and for defining the presence of a communication with the pancreatic ducts.

## Abbreviations

CA 19–9: carbohydrate antigen 19–9
CA125: carbohydrate antigen 125
CEA: carcino-embryonic antigen
CgA: chromogranin A
CT: computed tomography
EUS-FNA: endoscopic ultrasound fine needle aspiration
EUS-FNAC: endoscopic ultrasound fine needle aspiration citology
IPMN: intraductal papillary mucinous neoplasm
MCN: mucinous cystic neoplasm
MRCP: magnetic resonance cholangiopancreatography
NSE: neuron specific enolase
PFCs: pancreatic fluid collections
US: abdominal ultrasound
WON: walled-off necrosis
